# Effect of PRISMA 2009 on reporting quality in systematic reviews and meta-analyses in high-impact dental medicine journals between 1993–2018

**DOI:** 10.1371/journal.pone.0295864

**Published:** 2023-12-14

**Authors:** Kerry A. Sewell, Jana Schellinger, Jamie E. Bloss

**Affiliations:** 1 William E. Laupus Health Sciences Library, East Carolina University, Greenville, North Carolina, United States of America; 2 Center for Evidence-Based Policy, Oregon Health & Science University, Portland, Oregon, United States of America; Aga Khan University Hospital, PAKISTAN

## Abstract

**Introduction:**

The PRISMA guidelines were published in 2009 to address inadequate reporting of key methodological details in systematic reviews and meta-analyses (SRs/MAs). This study sought to assess the impact of PRISMA on the quality of reporting in the full text of dental medicine journals.

**Methods:**

This study assessed the impact of PRISMA (2009) on thirteen methodological details in SRs/MAs published in the highest-impact dental medicine journals between 1993–2009 (n = 211) and 2012–2018 (n = 618). The study further examined the rate of described use of PRISMA in the abstract or full text of included studies published post- PRISMA and the impact of described use of PRISMA on level of reporting. This study also examined potential effects of inclusion of PRISMA in Instructions for Authors, along with study team characteristics.

**Results:**

The number of items reported in SRs/MAs increased following the publication of PRISMA (pre-PRISMA: *M* = 7.83, *SD* = 3.267; post-PRISMA: *M* = 10.55, *SD* = 1.4). Post-PRISMA, authors rarely mention PRISMA in abstracts (8.9%) and describe the use of PRISMA in the full text in 59.87% of SRs/MAs. The described use of PRISMA within the full text indicates that its intent (guidance for reporting) is not well understood, with over a third of SRs/MAs (35.6%) describing PRISMA as guiding the conduct of the review. However, any described use of PRISMA was associated with improved reporting. Among author team characteristics examined, only author team size had a positive relationship with improved reporting.

**Conclusion:**

Following the 2009 publication of PRISMA, the level of reporting of key methodological details improved for systematic reviews/meta-analyses published in the highest-impact dental medicine journals. The positive relationship between reference to PRISMA in the full text and level of reporting provides further evidence of the impact of PRISMA on improving transparent reporting in dental medicine SRs/MAs.

## Introduction

Systematic reviews and meta-analyses play a critical role in informing point-of-care decisions, clinical guideline development teams, policy decisions, and directions for new research to fill gaps in knowledge. The importance and usefulness of systematic reviews and meta-analyses across stakeholder groups is reflected in their place at the top of the hierarchy of evidence as well as the high rates of citations for systematic reviews and meta-analyses (SRs/MAs) [1]. As such, the quality of the conduct and reporting of systematic reviews and meta-analyses has clinical, ethical, sociopolitical, and financial implications [2, 3]. The perceived and codified importance of systematic reviews and meta-analyses may also drive researchers to produce them without regard or sufficient attention to methodological and reporting quality, as indicated in studies of the increasing publication rates for systematic reviews and meta-analyses [4–8].

The methodological quality of systematic reviews and meta-analyses has been a long-standing concern [9–12], with considerable effort invested in creating and updating methodological guidelines and recommendations to improve rigor by organizations such as those from the Cochrane Collaboration and the Institute of Medicine, among others. Likewise, tools to assess methodological quality of published evidence syntheses, such as AMSTAR (A MeaSurement Tool to Assess systematic Reviews) [13] and risk of bias assessments for systematic reviews, such as ROBIS [14] were developed to assess published evidence syntheses. The early concerns about the methodological quality of SRs/MAs were quickly succeeded by concerns related to the reporting quality thereof, with the understanding that, without thorough and transparent reporting, neither methodological rigor nor reliability of results and recommendations could be assessed.

The concerns about the reporting quality of published evidence syntheses in the health fields led to the creation and dissemination of reporting guidelines for meta-analyses of randomized controlled trials, known as the Quality of Reporting of Meta-Analyses (QUORUM), in 1999 [15]. In 2009 the guideline was updated to include more criteria for reporting quality and renamed PRISMA (Preferred Reporting Items for Systematic reviews and Meta-Analyses) out of a need to address advances in the science of systematic reviews [16]. The PRISMA statement was updated again and republished with expanded reporting guidelines in March 2021 [17]. PRISMA extensions have also been created to guide reporting for specific review types and key pieces of reviews including abstracts, diagnostic test accuracy, equity, protocols, and searching, among others [18].

The QUORUM and PRISMA 2009 and 2020 guidelines were published in multiple, well-known English language journals (e.g., *PLOS Medicine*, *The Lancet*, *BMJ*) and translated into other languages. The PRISMA recommendations alone have been endorsed by many journals and publishers and have been widely cited [17]. Despite QUORUM and PRISMA 2009 being widely disseminated at the time of publication, studies of systematic reviews following the guidelines’ publication continued to note low methodological and reporting quality [19, 20]. The issues in methodological and reporting quality were not confined to specific clinical or disciplinary areas. Reviews of methodological and reporting quality in SRs and MAs from internal medicine [21], oncology [22], nursing [23], nephrology [24], rehabilitation [25], dental medicine [26, 27], and complementary and alternative medicine fields [28, 29] all note shortcomings in the conduct and reporting for systematic reviews.

The continued low reporting quality of SRs/MAs might be attributed to low levels of awareness of PRISMA, yet citation data from Scopus as well as a pooled analysis of studies evaluating uptake and impact of PRISMA indicate that it has wide acceptance and adoption, though imperfect adherence [30]. Many factors related to use of PRISMA guidelines may enable or inhibit use of the guidelines, such as study team size and primary author country of affiliation, with mixed results for both of these characteristics [31–33]. Likewise, the endorsement and requirement of adherence to PRISMA by disciplinary journals in Instructions for Authors (IA) is important and ought to prompt authors to improve their methodological reporting. However, the number of journals including PRISMA in IA is noted as being low, ranging between 12%-50%- of a given profession’s journals [23, 32, 34, 35].

While the inclusion of study-specific reporting guidelines (beyond SRs/MAs) in requirements or instructions for authors has been found to improve some reporting practices for primary research [36, 37], it is worth noting that research examining journals’ inclusion of PRISMA in IA, either as an endorsement or requirement, could not find sufficient evidence that there was a strong improvement in reporting [30, 38]. These same findings appear to be true for other reporting guidelines [38–40]. This may either be due to lack of clear instructions for peer reviewers on utilization of reporting guidelines during the peer review process, or authors’ openness to peer review comments related to needed improvements in reporting, or other pressures [41–44] Published evidence indicates that author uptake of the guidelines, evidenced through the statement of adherence to PRISMA within the abstract or full text of SRs/MAs, has a greater effect on reporting quality than the inclusion of PRISMA in IA [45].

Although studies elucidating reporting quality and methodological quality of SRs/MAs have been noted across the health disciplines for some time, the examination of these issues in dental medicine has only been a focus over the last decade, with greater focus on the methodological quality of SRs/MAs for this profession, rather than the reporting quality. Few studies have examined how well PRISMA reporting standards are adhered to within the full text of published SRs/MAs in dental medicine. The majority of studies assessing the impact of PRISMA on published dental medicine SRs/MAs focus on the effect of PRISMA for Abstracts (PRISMA-A) on the reporting quality of abstracts of systematic reviews [32, 46–50]. However, abstracts are so abbreviated that they cannot, for instance, provide a reproducible search strategy or fully describe selection processes, which, among other PRISMA checklist items, provide the critical information to allow the assessment of the methodological quality of the review and enable reproducibility and replicability.

Assessments of adherence to PRISMA in the full text of dental medicine SRs/MAs have utilized small sets of articles, though the articles sampled have never been those published in the highest impact journals for dental medicine [51]. The relationship between dental medicine journal inclusion of PRISMA in its IA is unknown. Strikingly, only a quarter (24.1%) of dental medicine journals in 2018 required adherence to PRISMA for published systematic reviews and meta-analyses [52]. In light of these lacunae in the literature, this study sought to improve the understanding of the effect of PRISMA 2009 on the reporting quality of the full text of SRs/MAs in the highest impact journals for dental medicine.

### Research questions

The authors sought to address four research questions related to the effect of PRISMA 2009 on reporting quality for SRs/MAs in Dental Medicine journals:

1. What was the effect of PRISMA 2009 on the level of reporting of key methodological details for systematic reviews and meta-analyses in high impact dental medicine journals?
2. Following the publication of the PRISMA 2009 Guidelines and Checklist, how frequently did authors in the selected dental medicine journals refer to PRISMA and what effect did described use of PRISMA have on reporting quality?
  1. How frequently did SRs/MAs in the selected, high-impact dental medicine journals refer to PRISMA 2009 in their abstracts or full-text?
  2. For authors referring to PRISMA, did they distinguish between PRISMA as a reporting guideline rather than a methodological guideline?
  3. Post-PRISMA, what effect did reference to PRISMA have upon the reporting quality of articles?
3. What effect do the characteristics of the study team (number of authors and country of primary author) have on adherence to PRISMA?
4. For high impact journals in dental medicine with recommendation or requirement of PRISMA 2009 in instructions for authors, was reporting quality higher?

## Materials and methods

### Data collection and sources

This study used published SRs/MAs from a compiled set of the twelve highest impact journals categorized as dental medicine journals in Journal Citation Reports (JCR) and Scimago (https://www.scimagojr.com/). The methods for determining the journals, searching for systematic reviews and meta-analyses within the selected journals, and assessing the resulting systematic reviews and meta-analyses are reported in detail in a previously published study [27] and supporting data and data documentation are available on the project’s Open Science Framework site [https://osf.io/hgkmq/].

Briefly, the top ten highest impact journals in the dental medicine categories from JCR and Scimago were combined and deduplicated, leaving a set of twelve journals (S1 File). SRs/MAs published in the twelve journals were identified via a targeted search in PubMed, run on June 18, 2018, available in S2 File. After the initial search was run, two authors (KS and JS) reviewed all the results to determine which records were legitimately systematic reviews or meta-analyses using a blinded screening method in Rayyan. Conflicting decisions about the records were resolved by consensus, after which 913 articles remained. Subsequent assessment of the size of the corpus of systematic reviews and meta-analyses published in journals classified as Dental journals in PubMed up to the date of October, 2018 indicates that the sample from the journals used for this study represents ~14% of the systematic reviews and meta-analyses for Dental journals included in PubMed published prior to 2019.

The articles from the selected journals were initially evaluated for key methodological and reporting characteristics using an evaluation instrument adapted from the instrument used by Rethlefsen et al. [21]. The evaluation instrument assessed the provision of details of blinding, study selection, and risk of bias, as well as search strategy inclusion in the included SRs and MAs. For the purposes of this study, additional data were added to the dataset from two sources: PubMed and Scopus. Additional data elements for this study include: the publication year for each record, specification of the study as a ‘systematic review’ or ‘meta-analysis’ in the title, number of authors, international collaboration of authors, country of the corresponding author and the first author, world region for those two positions (when they differed), reference to PRISMA in the abstract, reference to PRISMA in the full text, and whether the reference to PRISMA indicated use of PRISMA to guide reporting or conduct of the SR or MA. To add these data points, the complete set of PMIDs for the SRs and MAs in this sample were entered into PubMed, publication date information was downloaded and added to the existing dataset, and then a search combining the PMIDs with terms for systematic reviews or meta-analyses limited to the title field was run to find just the records meeting that PRISMA criterion. Additionally, within PubMed, Reference to PRISMA in abstracts was assessed using a search for PRISMA or “Preferred Reporting Items for Systematic Reviews and Meta-Analyses” in the abstract field of included records. Reference to PRISMA within the full text and the stated use of PRISMA to guide the methodological conduct or reporting was collected through assessment of the full text of the document. Additional details on these processes may be found on the Open Science Framework project space for this study.

The data related to number of authors, first and corresponding author country of affiliation, associated world region, along with presence/absence of international collaboration were all derived from publication data in Scopus. For author affiliations missing from the data provided by Scopus, author-supplied affiliations were determined from affiliation data from the author associated with publications from the same time period, along with general searches for scholarly profiles or public profiles for institutions for those authors, focusing on affiliation data for the time period of the publication. This data collection was performed by two authors (JB and KS) All first author countries were mapped to United Nations "Standard Country or Area Codes for Statistical Use" [53], along with the corresponding World Region and World SubRegion Codes from that publication.

Because the dataset for this study included articles published in high-impact dental medicine journals between 1993–2018, the PRISMA 2009 checklist was used to assess reporting quality. To determine how reporting items fit within the PRISMA 2009 requirements, the initial evaluation instrument’s assessment items are mapped to the PRISMA 2009 checklist items in Table 1, along with the additional datapoint of specification of SR or MA in the title. The authors note that several key methodological items were applicable to a single PRISMA item, namely for reporting eligibility criteria and study selection processes. The mapped items from the assessment instrument used in this study were kept separate to determine the extent of author reporting for those compound reporting items. It is also notable that several of these items are largely unchanged between the QUORUM, PRISMA 2009, and PRISMA 2020 checklists, such as the identification of the report as a systematic review in its titling, the specification of inclusion and exclusion criteria, and reporting study selection results for each phase of the review, though clarity for some of those items has improved between the reporting guidelines. Therefore, studies not meeting many of the assessed checklist items for PRISMA 2009 would typically fail to meet the same or similar reporting items for QUORUM and PRISMA 2020.

**Table 1:** Assessment instrument items mapped to 2009 PRISMA items.

PRISMA Item Number	Section/topic	Checklist Item Description	Assessment Items from Assessment Instrument
1	Title	Identify the report as a systematic review, meta-analysis, or both.	Title specifies systematic review or meta-analysis
6	Methods: Eligibility Criteria	Specify study characteristics (e.g., PICOS, length of follow-up) and report characteristics (e.g., years considered, language, publication status) used as criteria for eligibility, giving rationale.	Inclusion Criteria Reported Exclusion Criteria Reported
8	Methods: Search	Present full electronic search strategy for at least one database, including any limits used, such that it could be repeated.	Reproducible Search Provided
9	Methods: Study selection	State the process for selecting studies (i.e., screening, eligibility, included in systematic review, and, if applicable, included in the meta-analysis).	Explicit information about Use or Non-Use of Blinding Reported Number of People Reviewing Titles and Abstracts Number of People Reviewing Full Text
12	Methods: Risk of bias in individual studies	Describe methods used for assessing risk of bias of individual studies (including specification of whether this was done at the study or outcome level), and how this information is to be used in any data synthesis.	Reports risk of bias assessment
17	Results: Study selection	Give numbers of studies screened, assessed for eligibility, and included in the review, with reasons for exclusions at each stage, ideally with a flow diagram.	Number of Studies Found Reported Number of Duplicates Removed Reported Number of Titles/Abstracts Screened Reported Number of Full Text Screened Reported Number of Items Fully Included Reported

The publications from 2010–2011 were removed from the dataset to account for publication timelines for SRs/MAs written prior to or just following the publication of the 2009 PRISMA guidelines, as well as to account for a potential lag in awareness of PRISMA based upon diffusion of knowledge. Additionally, SRs/MAs affiliated with the Cochrane Organization were removed from the dataset. The removal of SRs and MAs published in 2010–2011, along with Cochrane-affiliated SRs/MAs left 829 articles for analysis. The process of selecting studies is outlined in the PRISMA flow diagram in S3 File. Variables were recoded for this study to allow for calculated variables (total number of PRISMA-mapped items met in each included publication) and for combining assessment items related to search strategy reporting. The refined, re-coded dataset, along with its data dictionary and data cleaning documentation are in the Open Science Framework project space for this study.

The websites for all journals selected for this study were examined to determine the inclusion of PRISMA in IA. This website survey was conducted between April and May, 2022. For all journals with PRISMA mentioned or required in IA, the journals were contacted to determine the date when PRISMA endorsement was added to IA. For journals not responding or providing insufficient information about the date of addition of PRISMA in IA, the Internet Archive (https://archive.org/web/) was used to attempt to access archived websites for those journals.

### Data analysis

All data were re-coded and analyzed in SPSS (version 28.0). For all items mapped to PRISMA items for this study, descriptive statistics were run. Crosstabs were run to assess the percent of SRs and MAs providing key methodological details before and after the publication of PRISMA. A Chi-square test of independence was run to determine the significance of differences between the groups and the effect size was determined using Cohen’s d. Significance was determined using p < 0.05.

For questions other than the first research question, this study analyzed the overall level of reporting for each publication using a calculated variable (total PRISMA-mapped items), derived from the sum of all key methodological detail items assessed for this study. This calculated sum for PRISMA-mapped items reported was used as the dependent variable when assessing the effect of author team characteristics on reporting, including author team size, international collaboration in author teams, and world region of first author. The sum total of PRISMA-mapped items was also used to assess the effect of described use of PRISMA in the abstract or full text on reporting level. For questions related to rate of inclusion of PRISMA in the abstract or full text of publications, as well as the described use of PRISMA in the full text, frequencies were run. The variables, maps to PRISMA items, statistical analysis methods chosen, and other key details are provided in Table 2.

**Table 2:** Data analysis.

Research Question	Associated Question(s) from Dataset	Independent Variable(s) (Code) with Response Code(s)	Dependent Variables(s) (Code) with Response Code(s)	Variable Type(s)	Analysis
What was the effect of PRISMA on the level of reporting of key methodological details for systematic reviews and meta-analyses in high impact dental medicine journals? Effect on overall number of items reported	Questions 16^a^, 17	Publication Prior to or After the Publication of PRISMA (PrePostPRISMA), 1 = Pre-PRISMA 2 = Post-PRISMA	Total PRISMA Items Met (PRISMAItemsMet), with a range of 0–13	Independent: Categorical/nominal; Dependent: Scale	Independent Samples t-Test
What was the effect of PRISMA on the level of reporting of key methodological details for systematic reviews and meta-analyses in high impact dental medicine journals? Effect on individual reporting items	Questions 3–15^b^; 17	Publication Prior to or After the Publication of PRISMA (PrePostPRISMA), 1 = Pre-PRISMA 2 = Post-PRISMA	For all variables listed below, 0 = No; 1 = Yes Did the authors list their inclusion criteria? (InclusionCriteriaListed) Did the authors list their exclusion criteria? (ExclusionCriteriaListed) Do the authors describe the use of blinding in the methodology? (BlindingYN) Do the authors report the number of people who reviewed the titles/abstracts in the review? (NumberPeopleScreenTiAb) Do the authors report the number of people who reviewed the full text of the initially included articles? (NumberPeopleScreenFT) Did the authors provide the number of studies found? (TotalSearchResultsReporting) Did the authors provide the number of duplicates removed? (DuplicatesRemovedReporting) Did the authors provide the number of titles/abstracts screened? (TiAbScreenNumberReporting) Did the authors provide the number of full text articles reviewed? (FullTextScreenNumberReporting) Did the authors provide the number of articles fully included? (FullIncludedArticlesNumberReporting) The authors report performing a risk of bias or quality assessment (RiskBiasAssessmentReported) Does the title identify the study as a systematic review or meta-analysis? (TitleSpecifiesSRorMA) Was a reproducible search was provided in the study? (ReproducibleSearchProvided)	Categorical/nominal	Crosstabs with Chi-Square
How frequently do SRs and MAs in the selected, high-impact dental medicine journals refer to PRISMA in their abstracts or full-text?	For items published after PRISMA (filter using Question 17) Questions 26, 27	For both variables listed below, 0 = No 1 = Yes; 99 = Not applicable (published prior to 2009) PRISMA in Abstract (PRISMA_AbstractText) PRISMA Described or Cited (PRISMADescribed_Cited)		Categorical/nominal	Frequencies
For authors referring to PRISMA, do they distinguish between PRISMA as a reporting guideline rather than a methodological guideline?	Dataset 3, Questions 28, 29	Authors’ described application of PRISMA within the text of the review (DescribedUsePRISMAFullText) 0 = Missing (did not mention use of PRISMA) 1 = Conduct (authors describe using PRISMA to guide the methodological conduct or performance of the study) 2 = Reporting (authors describe using PRISMA to guide the reporting or presentation of the review within the published report) 3 = Conduct and reporting (authors indicate that PRISMA was used to guide both the methodological design/conduct as well as the reporting of the review) 4 = Protocol development 5 = PRISMA only referenced in the title or description of the source of the flow diagram design 6 = In reference list but not explicitly discussed in the text 7 = Unclear application of PRISMA for the published manuscript 99 = Not applicable (published prior to 2009) Terminology Used When PRISMA application is unclear (TermsUsed_UnclearUse) 1 = adheres to 2 = adopts 3 = applies 4 = complies with 5 = conforms to 6 = considers 7 = follows 8 = in accordance with 9 = in line with 10 = organized using 11 = used/utilized		Categorical/nominal	Frequencies
Post-PRISMA, what effect does reference to PRISMA have upon the reporting quality of articles?	Questions 16, 28	Authors’ described application of PRISMA within the text of the review (DescribedUsePRISMAFullText) 0 = Missing (did not mention use of PRISMA) 1 = Conduct (authors describe using PRISMA to guide the methodological conduct or performance of the study) 2 = Reporting (authors describe using PRISMA to guide the reporting or presentation of the review within the published report) 3 = Conduct and reporting (authors indicate that PRISMA was used to guide both the methodological design/conduct as well as the reporting of the review) 4 = Protocol development 5 = PRISMA only referenced in the title or description of the source of the flow diagram design 6 = In reference list but not explicitly discussed in the text 7 = Unclear application of PRISMA for the published manuscript 99 = Not applicable (published prior to 2009)	Total PRISMA Items Met (PRISMAItemsMet), with a range of 0–13	Categorical/nominal (Independent); Scale (Dependent)	One-Way Analysis of Variance with Tukey post-hoc HSD
What effect do the characteristics of the study team have on adherence to PRISMA (for items published after PRISMA)? Total Number of Authors	Questions 16, 19	Total Number of Authors (TotalAuthors) Values: any whole number > 0;	Total PRISMA Items Met (PRISMAItemsMet), with a range of 0–13	Scale	Independent Samples t-Test
What effect do the characteristics of the study team have on adherence to PRISMA (for items published after PRISMA)? Author Team Sizes	Questions 16, 33	Total Number of Authors (TotalAuthors) Values: any whole number > 0; Total Authors by Study Team Size (TotalAuthorsRegrouped) 1 = 1–2 authors 2 = 3–6 authors 3 > 6 authors	Total PRISMA Items Met (PRISMAItemsMet), with a range of 0–13	Categorical/nominal (Independent); Scale (Dependent)	One-Way Analysis of Variance (author team size)
What effect do the characteristics of the study team have on adherence to PRISMA (for items published after PRISMA)? International collaboration or world region of first author	Questions 16, 25	International Collaboration (InternationalCollaboration), 0 = No 1 = Yes	Total PRISMA Items Met (PRISMAItemsMet), with a range of 0–13	Categorical/nominal (Independent); Scale (Dependent)	Independent Samples t-Test
What effect do the characteristics of the study team have on adherence to PRISMA (for items published after PRISMA)? World region of first author	Questions 16, 23	First Author World Region (FirstAuthorWorldRegion) 2 = Africa 9 = Oceania 19 = Americas 142 = Asia 150 = Europe	Total PRISMA Items Met (PRISMAItemsMet), with a range of 0–14	Categorical/nominal (Independent); Scale (Dependent)	One-Way Analysis of Variance (author team size)
For high impact journals in dental medicine with recommendation or requirement of PRISMA in instructions for authors, is reporting quality higher?	Questions 16, 30	PRISMA included in IA at time of publication (PRISMA_Instruction_Authors); 0 = No; 1 = Yes	Total PRISMA Items Met (PRISMAItemsMet), with a range of 0–13	Categorical/nominal (Independent); Scale (Dependent)	Crosstabs with Chi Square

^a^. This is a calculated variable derived from Questions 3–15.

^b^. All items here (Questions, 3–15) came from a Questionnaire (1 of 2) used in a previously published study. The variable names were re-coded and the values were recoded. Full documentation of variable name changes and recoding is provided in the codebook for this study.

## Results

### Characteristics of included SRs/MAs

SRs/MAs by year and journal: The number of SRs/MAs in the included journal set increased threefold between the time periods studied, with 211 SRs/MAs published between 1998–2009 (pre-PRISMA) and 618 published between 2012–2018 (post-PRISMA). The majority of the included records were published in the Journal of Clinical Periodontology, followed by Clinical Oral Implants Research and Journal of Dentistry.

Bibliometric data findings: For the purposes of this study, we assessed the number of authors per paper, the country, world region, and world sub-region of first authors, along with the country income classification, and the presence of international collaboration based upon author affiliation data. Our findings indicate that most SR/MA teams with publications in the selected high-impact dental medicine journals include 3–6 authors, with a mean number of 4.51. About one third of all included studies had international collaboration (35.9%).

First authors for the SRs/MAs were affiliated with institutions or dental practices primarily in Europe (n = 476, 57.4%), the Americas (n = 232, 28.0%, or Asia (n = 92, 11.1%). By world subregion, most authors were affiliated with institutions in Western Europe (n = 241, 29.1%), followed by Northern Europe (n = 137, 16.5%), and Northern America (*n* = 122, 14.7%). The vast majority of first authors were from institutions in high-income countries (n = 660, 79.6%) or upper-middle income countries (n = 163, 19.6%). Major characteristics of included studies are presented in Table 3. The full frequencies for world region, sub-region, country, and country income classification are in a S1 Table.

**Table 3:** Characteristics of included studies.

Publication characteristics	Published prior to PRISMA		Published after PRISMA		Full Sample	
	N	%	n	%	n	%
**Total authors**						
1	9	4.3%	0	0.0%	9	1.1%
2	40	19.0%	44	7.1%	84	10.1%
3	62	29.4%	114	18.4%	176	21.2%
4	50	23.7%	113	18.3%	163	19.7%
5	30	14.2%	142	23.0%	172	20.7%
6	12	5.7%	119	19.3%	131	15.8%
7	5	2.4%	47	7.6%	52	6.3%
8	2	0.9%	24	3.9%	26	3.1%
9	0	0.0%	9	1.5%	9	1.1%
10	0	0.0%	3	0.5%	3	0.4%
11	0	0.0%	0	0.0%	0	0.0%
12	1	0.5%	3	0.5%	4	0.5%
**International collaboration**						
Yes	55	26.1%	243	39.3%	298	35.9%
No	156	73.9%	375	60.7%	531	64.1%
**First Author World Region**						
Europe	152	72.0%	324	52.4%	476	57.4%
Americas	41	19.4%	191	30.9%	232	28.0%
Asia	9	4.3%	83	13.4%	92	11.1%
Oceania	8	3.8%	18	2.9%	26	3.1%
Africa	1	0.5%	2	0.3%	3	0.4%

*Note*. There were 211 included systematic reviews and meta-analyses published prior to the publication of PRISMA guidelines and 618 systematic reviews and meta-analyses in the selected journals between 2012 through the date of the last search in 2018, for a full sample size of 829.

### What was the effect of PRISMA 2009 on the level of reporting of key methodological details for systematic reviews and meta-analyses in high impact dental medicine journals?

There was a statistically significant difference in the number of key methodological details reported among articles published after 2011 (*M* = 10.55, *SD* = 1.4) as compared to those published prior to the release of PRISMA 2009 (*M* = 7.83, *SD* = 3.267); *t*(827) = -12.915, *p* < .001. Examining the individual methodological reporting items tracked, crosstabs with Chi-Square revealed that reporting of each individual item improved following publication of PRISMA in 2009. Results of the crosstabs with Chi-Square are presented in Table 4.

**Table 4:** Reporting by methodological item, pre- and post-PRISMA 2009.

Reporting Item	Published prior to PRISMA				Published after PRISMA				df	Asymptotic Significance (2-sided)
	Reported		Not Reported		Reported		Not Reported			
	n	%	n	%	n	%	n	%		
Title Specifies Systematic Review or Meta-Analysis	157	74.4%	54	25.6%	549	88.8%	69	11.2%	1	< .001
Inclusion Criteria Listed	192	91.0%	19	9.0%	599	96.9%	19	3.1%	1	< .001
Exclusion Criteria Listed	143	67.8%	68	32.2%	485	78.5%	133	21.5%	1	0.002
Reproducible Search Provided	70	33.2%	141	66.8%	356	57.6%	262	42.4%	1	< .001
Report Number of People Screening Titles and Abstracts	122	57.8%	89	42.2%	450	72.8%	168	27.2%	1	< .001
Report Number of People Screening Full Text	121	57.3%	90	42.7%	454	73.5%	164	26.5%	1	< .001
Blinding Procedures Described	129	61.1%	82	38.9%	477	77.2%	141	22.8%	1	< .001
Total Initial Search Numbers Reported	152	72.0%	59	28.0%	588	95.1%	30	4.9%	1	< .001
Number of Duplicates Removed Reported	12	5.7%	199	94.3%	328	53.1%	290	46.9%	1	< .001
Number of Titles and Abstracts Screened Reported	133	63.0%	78	37.0%	572	92.6%	46	7.4%	1	< .001
Number of Full Texts Screened Reported	143	67.8%	68	32.2%	576	93.2%	42	6.8%	1	< .001
Number of Fully Included Articles Reported	192	91.0%	19	9.0%	607	98.2%	11	1.8%	1	< .001
Risk of Bias Assessment Reported	87	41.2%	124	58.8%	478	77.3%	140	22.7%	1	< .001

### Following the publication of the PRISMA 2009 guidelines and checklist, how frequently did authors in the selected dental medicine journals refer to PRISMA and what effect did described use of PRISMA have on reporting quality?

#### How frequently did SRs and MAs in the selected, high-impact dental medicine journals refer to PRISMA in their abstracts or full-text?

Among SRs/MAs published after 2011, PRISMA is rarely mentioned in the abstracts of author-identified SRs/MAs in the highest-impact dental medicine journals. For the journals assessed, over 90% of the abstracts did not mention PRISMA, with only 8.9% explicitly describing use of PRISMA in the abstract for the published SR/MA. Within the full text of the SR/MAs published after 2011, authors explicitly mention PRISMA in the manuscript body in roughly 60% of publications (n = 370, 59.87%). The examination of the reference list to identify studies that referenced PRISMA but did not name it explicitly in the manuscript body, does not greatly improve the number of publications in which authors indicate knowledge of PRISMA (n = 376, 60.84% of sample). Data provided in Table 5.

**Table 5:** Authors’ explicit description of the use of PRISMA in dental medicine systematic reviews, 2012–2018.

Primary Publication Component	Explicitly Mention PRISMA		Omit to Mention PRISMA	
	*n*	*%*	*n*	*%*
**Abstract**	55	8.9	563	91.1
**Full Text (Main body of manuscript only)**	370	59.87	248	40.13

#### For authors referring to PRISMA, did they distinguish between PRISMA as a reporting guideline rather than a methodological guideline?

The intended application of PRISMA is not well reflected in the described use of PRISMA within dental medicine SR/MAs that mention the guidelines. Of the articles that mentioned or cited PRISMA, the guidance was most frequently described as being used to guide the methodological conduct of the study (35.6%), followed by its correct application as a guideline to improve the reporting of the SR/MA being described in the publication (27.4%). In other cases, the guidelines were referenced as guiding both conduct and reporting (6.6%) or protocol development (9.6%). The authors note that the application of PRISMA was unclear for 15.4% of the articles assessed for this study. The descriptive statistics, including the language used to describe the use of PRISMA for those publications coded as ‘unclear,’ is presented in Table 6.

**Table 6:** Authors’ described Use of PRISMA within published dental medicine systematic reviews and meta-analyses, 2012–2018.

Described Use of PRISMA within the full text	*n*	*%*
PRISMA not mentioned or referenced anywhere	242	39.2
Guide for conduct	134	21.7
Guide for reporting	103	16.7
Guide for both conduct and reporting	25	4.0
Guide for protocol development^a^	36	5.8
Only mentioned in the titling/description of the included flow diagram	14	2.3
In reference list only	6	1.0
Application unclear based upon language in text	58	9.4
Study utilized PRISMA	29	4.7
Study organized using PRISMA	6	0.97
Study in line with PRISMA	6	0.97
Study in accordance with PRISMA	4	0.65
Study follows PRISMA	4	0.65
Study considers PRISMA	3	0.49
Study conforms to PRISMA	2	0.32
Study complies with PRISMA	1	0.16
Study applies PRISMA	1	0.16
Study adopts PRISMA	1	0.16
Study adheres to PRISMA	1	0.16

#### Post-PRISMA, what effect did reference to PRISMA in the full text have upon the reporting quality of articles?

For SR/MA articles published post-PRISMA, a one-way ANOVA revealed there was a significant effect of description of PRISMA application within the full text on the number of items meeting PRISMA reporting requirements [*F*(7,610) = 13.81, *p* < .001]. The results of the One-Way ANOVA are presented in Table 7 and means and standard deviations for number of items reported by described use of PRISMA are presented in Table 8. Post hoc comparisons using the Tukey HSD indicated that any explicitly described application of PRISMA is significantly associated with improved reporting of the key methodological items tracked in this study compared to articles not explicitly describing the application of PRISMA to the SR/MA process (*M* = 9.55, *SD* = 2.867); *t*(616) = -9.5, *p* < .001. There was a non-significant difference between the absence of PRISMA in the full text and only mentioning PRISMA through the titling of the flow diagram as a PRISMA flow diagram (*p* = 1.0) or use of PRISMA guidelines as a supporting reference without explicit naming of the guidelines in the full text (*p* = .99). Likewise, there was a non-significant difference between authors explicitly describing the guidelines as being used for the conduct of the study (*p* = .98), the reporting of it (*p* = 1.0), both conduct and reporting (*p* = 1.0), protocol development (*p* = 1.0), or an unclear application of PRISMA (*p* = 1.0). Taken together, the results indicate that any explicit description of PRISMA in the full text improves the level of reporting of items required by PRISMA.

**Table 7:** One-Way analysis of variance of PRISMA item reporting by use of PRISMA 2009 within the full text of published manuscript.

Source	*df*	*SS*	*MS*	*F*	*p*
Between Groups	7	479.349	68.478	13.806	< .001
Within Groups	610	3025.694			
Total	617	3505.044			

**Table 8:** Descriptive statistics for PRISMA reporting based upon described application of PRISMA 2009 in article full text.

Described PRISMA Application within Full Text	*Number of Items Reported*	
	*M*	*SD*
PRISMA not mentioned at all	9.50	2.90
Guide for Conduct	11.16	1.76
Guide for Reporting	11.44	1.48
Guide for both Conduct and Reporting	11.24	1.05
Guide for Protocol Development	11.19	1.39
Only mentioned in the titling/description of the included flow diagram	9.86	2.41
In reference list only	10.33	3.20
Application unclear based upon language in text	11.43	1.56

### What effect do the characteristics of study team members have on adherence to PRISMA?

#### Does the number of authors on a study team increase the number of key methodological details reported?

There was a statistically significant difference in the number of key methodological items reported based upon the number of authors involved in the study, as determined by one-way ANOVA. This was true for all of the SR/MAs sampled for this study (*F*(2,826) = 35.544, *p* < .001), as well as for the set of those in the sample published post-PRISMA (*F*(2,615) = 11.235, *p* < .001)). For all studies in the sample (regardless of publication date), a Tukey post-hoc test revealed that the number of key methodological items reported was significantly higher for mid-sized study teams (3–6 authors; *M* = 10.10, *SD* = 2.74) and large study teams (>6 authors; *M* = 10.44, *SD* = 2.54) than for very small teams (< 3 authors; *M* = 7.59, *SD* = 3.23). Limited to just the studies published post-PRISMA, the Tukey post-hoc test revealed that this significant difference in the number of items reported held true for mid-sized study teams (*M* = 10.67, *SD* = 2.32) compared to either very small study teams (*M* = 9.02, *SD* = 2.73) or larger study teams (*M* = 10.62, *SD* = 2.34). There was a non-significant difference between the number of key methodological items reported by mid-sized study teams and large study teams.

#### Does international collaboration or the world region of first authors have an effect on the number of key methodological items reported?

To understand the effect of study team characteristics on the level of compliance with PRISMA in the published manuscript, a linear regression was run. The results of the linear regression revealed that neither international collaboration nor the world region of first authors were statistically significant predictors to the model (p> 0.5).

### Assessing the predictors of levels of reporting of key methodological details

To understand the impact of total number of authors and mention of PRISMA 2009 in the full text, while controlling for the other variables tested (total authors, mentioning PRISMA in the abstract, international collaboration, and world region of first author) on the level of transparent reporting for dental medicine SRs from 2011–2018, a linear regression analysis was run. The overall regression was statistically significant (R2 = 13.9, *F*(8, 609) = 12.24, *p* < .001). The results of the linear regression indicate that the total number of authors and mention of PRISMA in the full text account for 13.9% of the variation in the number of key methodological items reported. This means that 86.4% of the variation in key methodological items correctly reported cannot be explained by the number of authors or direct mention of PRISMA alone. The results of the linear regression are reported in Table 9.

**Table 9:** Regression analysis summary for predictors of number of key methodological items reported (post-PRISMA).

Variable	B	95% CI	β	t	p
Constant	8.987	[8.398 9.575]		29.98	< .001
International Collaboration	-0.196	[0.169 0.975]	-0.04	-1.05	0.29
Total Authors	0.125	[0.02 0.229]	0.09	2.35	0.02
PRISMA mentioned in the Abstract	0.359	[-.284 1.00]	0.04	1.10	0.27
PRISMA explicitly mentioned in the Full Text	1.716	[1.34 2.09]	0.35	8.94	< .001
First Author Affiliation in Africa	0.210	[-2.90 3.32]	0.01	0.13	0.993
First Author Affiliation in Americas	-0.204	[-0.61 0.68]	-0.04	-1.00	0.894
First Author Affiliation in Asia	0.132	[-0.41 0.68]	0.02	0.48	0.891
First Author Affiliation in Europe (reference value)	-0.210	[-3.317 2.897]	-0.04	-0.13	0.900
First Author Affiliation in Oceania	0.272	[-0.79 1.34]	0.02	0.50	0.969

### For high impact journals in dental medicine with recommendation or requirement of PRISMA in instructions for authors, is reporting quality higher?

#### PRISMA in IA

Five of the journals responded with precise information about the date when PRISMA was added to their IA; in only one instance was PRISMA part of IA prior to 2018 among the selected journals (*Clinical Oral Implants Research*, which added PRISMA in IA in March, 2017). Webpages for all other journals either did not recommend or mention PRISMA in their IA, or the website content was insufficiently captured in the Internet Archive to allow for determination of inclusion of PRISMA in IA in prior iterations of the IA page on the journals’ websites. In other cases, journal editors responded with statements about adoption of PRISMA as a principle without it being a requirement in IA. Due to the overly small sample size for analysis, the insufficient digital archiving of journals’ instructions for authors, and the date of the search for articles assessed in this study, the planned analysis of the effect of inclusion of PRISMA in IA on reporting quality could not be undertaken.

## Discussion

Although the QUORUM statement and checklist were published nearly a decade prior to the release of PRISMA in 2009, the PRISMA guidelines have served as a more widely recognized set of reporting guidelines for the exponentially increasing number of systematic reviews published across the disciplines. They are intended to serve the critical purpose of providing sufficient information to allow readers to assess the methodological quality of the conduct of the SR/MA and decide if the findings are trustworthy for the purposes of clinical, policy, or personal health decisions. As Davidoff [54, p. 231] notes, “accurate and transparent reporting is like turning the light on before you clean up a room: It doesn’t clean it for you but does tell you where the problems are.” This is increasingly important as umbrella reviews (including meta-umbrella reviews) are undertaken to better understand the benefits and harms of an array of interventions or exposures. Low quality reporting in the systematic reviews and meta-analyses meeting inclusion criteria in the umbrella then create additional difficulties for the team involved in the umbrella review.

The development and publication of high-quality reporting guidelines is the critical first step to addressing sub-optimal reporting quality. However, in order for reporting issues to be addressed, authors must be aware of and understand the correct application of those reporting guidelines. Only through dissemination of reporting guidelines, paired with author, editor, and peer reviewer education on how to use them, can reporting issues in studies be improved. These same observations have been made in the assessment of the impact of other reporting guidelines, such as the ARRIVE reporting guidelines [39, 55]. This study sought to assess the impact of PRISMA guidelines on the level of reporting of a subset of key methodological details in SRs/MAs in high-impact dental medicine journals. In assessing the frequency of described use of PRISMA in the full text of articles published after 2011, it also provides something of a measure of awareness and uptake of the guidelines, though awareness and utilization of guidelines may not track neatly with one another.

The study findings indicate that, following the widespread dissemination of PRISMA 2009 guidelines, there was a statistically significant improvement in the number of key methodological details in the SRs/MAs in the set of high-impact dental medicine journals we sampled. Although there are no other studies examining whether the PRISMA guidelines improved methodological reporting in dental medicine as a whole, one study has examined the effect of PRISMA on the reporting quality of systematic reviews in orthodontics [31]. Researchers note that the search strategies and selection processes in dental medicine SRs are generally not reproducible [26, 27]. Limited studies have examined whether PRISMA improved reporting in other health-related fields. Our results are consistent with those from the fields of pain medicine [56], psychology [57], nursing [58], vascular surgery [59], and radiology [60]. Our results are also consistent with studies indicating that PRISMA for Abstracts has improved the quality of dental medicine abstracts in SRs since it was published [61, 62].

Our assessment of the mention of PRISMA in the abstract and full text of the SRs/MAs indicate that PRISMA is explicitly mentioned in the full text of SRs/MAs far more frequently than in abstracts (59.9% vs. 8.9%, respectively). This may be due to word count limits for the journals selected, an issue which inhibits the reporting of methodological information for all study types. It is also far less critical for PRISMA to be mentioned in abstracts than in full texts. While PRISMA was explicitly mentioned in the full text of almost 60% of the SRs/MAs sampled, an assessment of the language used to describe its application indicates that authors’ textually expressed awareness of PRISMA and their understanding of the intended application of those guidelines are not necessarily aligned. Less than a quarter (21.7%) of the published SRs/MAs published after 2011 correctly referred to the guidelines as guiding the reporting of the review. While there is a relationship between the conduct of a study and its record [37], the PRISMA Statement is clear in its intended application as guiding the reporting of methodological details rather than the methodological conduct of the review. There are key differences in these two considerations. For example, while the guidelines’ requirement to report risk of bias assessment details may cue authors to undertake risk of bias assessment for included studies, that cue does not guarantee that the risk of bias assessment instrument was appropriately chosen or properly applied. Likewise, a reproducible search may neither be rigorous in its conceptualization, nor appropriately mapped across relevant databases. Such methodological quality assessments are better determined through assessment instruments such as AMSTAR [13] or ROBIS [14], and are the considerations that should ideally, ultimately drive the level of trust in the results and recommendations of the SR/MA. In spite of the apparent misunderstanding of the intended application of PRISMA, the authors note that articles that explicitly mentioned PRISMA in the full texts assessed improved reporting, even when authors described PRISMA as having guided the conduct of the SR/MA.

The study also assessed the effect of author team characteristics on the level of adherence to PRISMA for the selected reporting items. Our findings indicate that the number of authors involved is the sole author team factor associated with improved adherence to PRISMA, with mid-sized and large study teams having a higher number of items reported than small teams. One possible reason is that a larger team brings together people with different skill sets and experience levels, and that the chances of a larger team having someone who is well-versed at reporting methodology and/or being familiar with PRISMA are higher. The lack of correlation between first author world region or international collaboration with level of key methodological items reported indicates that the linguistic and geographic characteristics of teams matter far less than those individuals’ awareness of PRISMA. This is notable, given that the guidelines are primarily in English, with limited translations for other languages. Although the country classification by income of first authors was coded for the dataset, this study did not assess how the country income classification of first authors might influence the level of adherence to PRISMA. The inclusion of that data point did, however, indicate that SRs/MAs in the selected journals are primarily authored by those in high-income or upper-middle income countries. The ramifications of that are outside of the scope of this review but worth further study.

The lack of inclusion of PRISMA in the IA for the set of journals sampled in this study as of spring 2021, is of note. The ideal is certainly for authors to be aware of the reporting guidelines applicable to the type of study their manuscript reports, but IA have a potential to address awareness gaps by explicitly stating that SRs/MAs submitted must adhere to PRISMA and provide the checklist along with the manuscript. Panic, et al., noted that there is evidence that these recommendations improve the reporting of SR/MA methodology. As such, and out of a concern related to broader reproducibility issues, many journals have adopted the practice of recommending or requiring reporting that follows PRISMA guidelines for publication. Among dental medicine journals, Ruy Carneiro et al. [52] noted that only a quarter of journals in the field required adherence to PRISMA for published systematic reviews and meta-analyses. That said, this recommendation is only effective if journals and peer reviewers choose to enforce the recommendations and turn away articles that do not follow PRISMA reporting guidelines. Peer reviewers who are well-versed in PRISMA and SR methodology should be selected to review SRs and MAs.

Although this study found larger author team sizes and explicitly described use of PRISMA in the full text improved the level of reporting of the key methodological items in published SRs/MAs, it only explained about 14% of the variability in the number of items correctly reported. The authors conclude that the awareness of the guidelines and an understanding of each reporting item must ultimately come down to the level of education about the reporting guideline among the individuals involved in the SR/MA. This same need to promote meaningful implementation of reporting guidelines will remain true for PRISMA 2020. Previous studies note the critical role of librarians and statisticians in review teams for adequate reporting of methodological details within their respective purview [21, 27, 63, 64].

It is also notable that many of the reporting items are complex and compound, such as the requirement to provide a reproducible search. For example, although authors may provide a search, an understanding of what makes a search reproducible would require more extensive training in search syntax, along with the record-keeping required. Likewise, details related to selection processes ask authors to describe any automation tools used in study selection processes; authors may not assume that deduplication features or screening artificial intelligence models built into review software would fall into the category of automation tools needing clear description. These issues are likely yet more difficult for authors faced with the increased level of search reporting required in the PRISMA 2020 statement. The PRISMA 2020 Statement and PRISMA-S extension address some of the shortcomings noted in study selection and search processes, respectively. Authors would ideally utilize the Explanation and Elaboration publications to better understand the meaning and best practices attached to checklist items.

### Limitations

We only selected the top dental medicine journals, and they may not be representative of all journals in the field of dental medicine. These results likely are not generalizable to SRs and MAs in all healthcare fields. We removed articles from 2010–2011 to account for publication timelines for SRs and MAs written prior to or just following the publication of the 2009 PRISMA guidelines, as well as to account for a potential lag in awareness of PRISMA based upon diffusion of knowledge. We cannot, however, determine exactly when authors became aware of PRISMA, nor if the authors read the Explanation and Elaboration of PRISMA 2009, rather than simply employing the checklist. We also cannot determine if their understanding of PRISMA as a reporting guideline was correct, even when described as such; likewise, authors describing PRISMA as having guided the conduct of the review may have employed it as a reporting guideline. Because of the publication dates for the studies assessed, our study used the PRISMA 2009 checklist as its reference point for reporting items. As previously noted, many of the items assessed are included in the QUORUM, PRISMA 2009 and PRISMA 2020 checklists. A follow-up study to assess improvements in reporting for items uniquely, expressly required in PRISMA 2020 would help further elucidate awareness and meaningful use of PRISMA 2020 among authors of SRs/MAs.

In considering geographic factors related to author teams, this study only assessed affiliation information for the first author. Authorship is a complex issue and while a first author is assumed to bear the greatest responsibility for the study that is reported, if all authors earn their authorship, then all authors contribute to the study and the resulting manuscript. Therefore, only assessing the first author’s characteristics may overly simplify team characteristics. We also note that the first author’s country, world region, or world sub-region as the geographic units of assessment may be overly reductive. The author’s specific institution–its level of funding and research engagement–or the author’s alma mater, mentor, or individual interest in reporting within frameworks of reproducibility may all have an effect on their use and understanding of PRISMA and similar reporting guidelines. These are factors upon which broad geographic units may have limited bearing. We did not account for these factors, many of which would elude measurement using current affiliation data or author profile services.

Many of the items we examined may also have improved due to the development of software that facilitates the review process, keeping track of data points typically included in a flow diagram, such as numbers of records at each stage of the screening process. Our study did not examine the full text of included articles to track if authors described using software such as Rayyan, Covidence, or equivalents. Studies of the effect of dedicated review software on some of the reporting items in PRISMA would be a worthwhile addition to the literature.

Finally, we were unable to determine exactly when individual journals included in this study recommended or required authors to report their methods based on PRISMA guidelines. We note that, of the journals that we examined and that responded to requests for information on the date PRISMA was added to IA, only one had PRISMA as part of the IA prior to 2018 and noted that it was initially only a recommendation rather than a requirement. This is a limitation based upon the journals selected for the study. An analysis of compliance with PRISMA reporting guidelines, based on individual IA across a broader range of dental medicine journals, stratified by IA recommending use of PRISMA versus requiring use of PRISMA, could further enlighten this discussion about the impact of PRISMA in IA.

## Conclusion

While conduct and reporting of research are distinct concepts, they are closely intertwined for systematic reviews [2]. This study of the implementation of PRISMA 2009 reporting guidelines in dental SRs and MAs indicates that there is a need for increased use of and education related to PRISMA, particularly following the publication of PRISMA 2020. We suggest that those who are conducting SRs in dental medicine should become familiar with the PRISMA 2020 Statement in order to understand the complex nature of the individual reporting items and the methodological concerns informing the reporting items, *prior* to the start of the review process. Author examination of the Explanation and Elaboration publication accompanying PRISMA 2020 [65] is highly recommended, beyond just using the checklist as a standalone item. The employment of methodological guidelines for SRs/MAs might also help elucidate the meaning and importance of the reporting items. We further suggest that study teams should become familiar with the PRISMA extensions that apply to their reviews. We believe that the requirement by journals for SR teams to comply with PRISMA will improve methodological reporting in SRs, but that journals should either select peer reviewers who are trained in PRISMA to review submitted SRs/MAs or provide training materials on applicable reporting guidelines for peer reviewers less familiar with those guidelines. In an ideal world, requiring peer reviewers to assess manuscripts against applicable reporting guidelines, along with the methodological and conceptual merit of the submission, would address some shortcomings in reporting. We continue to advocate for librarians and information specialists to be fully-credited members of the study team, as many have been trained in PRISMA requirements. The results of this study indicate forward progress toward having rigorous and reproducible SRs/MAs, but areas for improvement are clear as well, and we believe these actions will improve research products in dental medicine. We further believe that these actions will improve reporting for SRs/MAs in all other areas of healthcare.

## Supporting information

S1 File Journals included in review and respective Scimago journal rank. (DOCX)

S2 File Search strategy for PubMed. Search strategy to identify systematic reviews and meta-analyses, mapped to PubMed syntax and controlled vocabulary.
(DOCX)

S3 File Flow diagram for selection of published SRs/MAs analyzed in this study. (DOCX)

S1 Table First authors’ affiliations by world region, sub-region, and country, along with income classification. Provides data on the geographic location of first authors, using affiliation data from author profiles, and mapped to UN M49 codes, along with the income classification for the first author’s country of affiliation.
(DOCX)
