# Supplementary material for: Effect of PRISMA 2009 on reporting quality in systematic reviews and meta-analyses in high-impact dental medicine journals between 1993–2018
Source: PLoS One. 2023 Dec 14;18(12):e0295864. doi: 10.1371/journal.pone.0295864 (PMC10721095; doi:10.1371/journal.pone.0295864)
Supplement: S1 File — (DOCX) [file pone.0295864.s001.docx]

**S1 Journals**

**Journals included in review and respective Scimago Journal Rank (from scimagojr.com)**

| **Journal Name** | **Scimago Journal Rank in August, 2023** |
| --- | --- |
| Periodontology 2000 | 3.457 |
| International Journal of Oral Science | 2.603 |
| Journal of Clinical Periodontology | 2.407 |
| Journal of Dental Research | 1.872 |
| European Journal of Oral Implantology | 1.788 |
| Clinical Oral Implants Research | 1.691 |
| International Endodontic Journal | 1.506 |
| Dental Materials | 1.283 |
| Oral Oncology | 1.271 |
| Journal of Periodontology | 1.270 |
| Journal of Dentistry | 1.189 |
| Monographs in oral science | 0.53 |
