# Supplementary material for: Effect of PRISMA 2009 on reporting quality in systematic reviews and meta-analyses in high-impact dental medicine journals between 1993–2018
Source: PLoS One. 2023 Dec 14;18(12):e0295864. doi: 10.1371/journal.pone.0295864 (PMC10721095; doi:10.1371/journal.pone.0295864)
Supplement: S3 File — (DOCX) [file pone.0295864.s003.docx]

**S3 PRISMA Flow**

**Flow Diagram for selection of published SRs/MAs analyzed in this study**

**Identification of studies via databases**

Records removed *before screening*:

Duplicate records removed (n = 1)

Records identified from journal-specific search in PubMed:

Databases (n = 1344)

**Identification**

Records screened

(n = 1343)

Records excluded

(n = 429)

Reports sought for retrieval

(n = 913)

**Screening**

Reports assessed for eligibility

(n = 913)

Reports excluded:

Published between 2010-2011 (n = 76)

Cochrane organization involved in review (n = 8)

Studies included in review

(n = 839)

**Included**

*From:*  Page MJ, McKenzie JE, Bossuyt PM, Boutron I, Hoffmann TC, Mulrow CD, et al. The PRISMA 2020 statement: an updated guideline for reporting systematic reviews. BMJ 2021;372:n71. doi: 10.1136/bmj.n71

For more information, visit: <http://www.prisma-statement.org/>
