# Supplementary material for: Effect of PRISMA 2009 on reporting quality in systematic reviews and meta-analyses in high-impact dental medicine journals between 1993–2018
Source: PLoS One. 2023 Dec 14;18(12):e0295864. doi: 10.1371/journal.pone.0295864 (PMC10721095; doi:10.1371/journal.pone.0295864)
Supplement: S1 Table — Provides data on the geographic location of first authors, using affiliation data from author profiles, and mapped to UN M49 codes, along with the income classification for the first author’s country of affiliation. (DOCX) [file pone.0295864.s004.docx]

**S4 Table.**

**First authors’ affiliations by world region, sub-region, and country, along with income classification**

| **World Region** | **World Sub-region** | **Country** | **n** | **%** | **Country Classification by Income** |
| --- | --- | --- | --- | --- | --- |
| Europe | | |  |  |  |
|  | Western Europe | |  |  |  |
|  |  | Switzerland | 71 | 8.6% | High-Income |
|  |  | Germany | 65 | 7.8% | High-Income |
|  |  | Netherlands | 64 | 7.7% | High-Income |
|  |  | Belgium | 18 | 2.2% | High-Income |
|  |  | France | 12 | 1.4% | High-Income |
|  |  | Liechtenstein | 6 | 0.7% | High-Income |
|  |  | Austria | 5 | 0.6% | High-Income |
|  | Northern Europe | |  |  |  |
|  |  | United Kingdom | 81 | 9.8% | High-Income |
|  |  | Sweden | 38 | 4.6% | High-Income |
|  |  | Denmark | 7 | 0.8% | High-Income |
|  |  | Iceland | 4 | 0.5% | High-Income |
|  |  | Lithuania | 3 | 0.4% | High-Income |
|  |  | Ireland | 2 | 0.2% | High-Income |
|  |  | Finland | 1 | 0.1% | High-Income |
|  |  | Norway | 1 | 0.1% | High-Income |
|  | Southern Europe | |  |  |  |
|  |  | Italy | 64 | 7.7% | High-Income |
|  |  | Spain | 20 | 2.4% | High-Income |
|  |  | Greece | 11 | 1.3% | High-Income |
|  |  | Serbia | 1 | 0.1% | Upper-Middle Income |
|  | Eastern Europe | |  |  |  |
|  |  | Czech Republic | 2 | 0.2% | High-Income |
| Americas | | |  |  |  |
|  | Northern America | |  |  |  |
|  |  | United States | 109 | 13.1% | High-Income |
|  |  | Canada | 13 | 1.6% | High-Income |
|  | Latin America and the Caribbean | |  |  |  |
|  |  | Brazil | 100 | 12.1% | Upper-Middle Income |
|  |  | Colombia | 6 | 0.7% | Upper-Middle Income |
|  |  | Chile | 3 | 0.4% | High-Income |
|  |  | Argentina | 1 | 0.1% | Upper-Middle Income |
| Asia | | |  |  |  |
|  | Eastern Asia | |  |  |  |
|  |  | China | 51 | 6.2% | Upper-Middle Income |
|  |  | Hong Kong | 10 | 1.2% | High-Income |
|  |  | Taiwan | 9 | 1.1% | High-Income |
|  |  | Japan | 4 | 0.5% | High-Income |
|  |  | Republic of Korea | 1 | 0.1% | High-Income |
|  | Western Asia | |  |  |  |
|  |  | Saudi Arabia | 5 | 0.6% | High-Income |
|  |  | Israel | 2 | 0.2% | High-Income |
|  |  | Jordan | 2 | 0.2% | Upper-Middle Income |
|  |  | Iran | 1 | 0.1% | Lower-Middle Income |
|  |  | Turkey | 1 | 0.1% | Upper-Middle Income |
|  |  | Yemen | 1 | 0.1% | Lower Income |
|  | South-eastern Asia | |  |  |  |
|  |  | Singapore | 3 | 0.4% | High-Income |
|  |  | Malaysia | 1 | 0.1% | Upper-Middle Income |
|  | Southern Asia | |  |  |  |
|  |  | Sri Lanka | 1 | 0.1% | Lower-Middle Income |
| Oceania | | | 26 | 3.1% |  |
|  | Australia and New Zealand | |  |  |  |
|  |  | Australia | 20 | 2.4% | High-Income |
|  |  | New Zealand | 6 | 0.7% | High-Income |
| Africa | | |  |  |  |
|  | Northern Africa | | 2 | 0.2% |  |
|  |  | Morocco | 1 | 0.1% | Lower-Middle Income |
|  |  | Egypt | 1 | 0.1% | Lower-Middle Income |
|  | Southern Africa | |  |  |  |
|  |  | South Africa | 1 | 0.1% | Upper-Middle Income |

*Note*. Country classifications by world region and sub-region are from the U.N.’s *"Standard Country or Area Codes for Statistical Use"*. The income classifications are from the World Bank *Country and Lending Groups* classification for 2023.
