# Supplementary material for: Effect of PRISMA 2009 on reporting quality in systematic reviews and meta-analyses in high-impact dental medicine journals between 1993–2018
Source: PLoS One. 2023 Dec 14;18(12):e0295864. doi: 10.1371/journal.pone.0295864 (PMC10721095; doi:10.1371/journal.pone.0295864)
Supplement: Attachment — Submitted filename: Response_To_Reviewers.docx [file pone.0295864.s005.docx]

Dear Dr. Umer,

We thank the reviewers for their thoughtful comments and detail our responses (in italics) to each of the concerns below. We believe responding to these concerns has strengthened the manuscript.

**Reviewer #1:**

I read the manuscript yesterday, but didn't get very far. The manuscript is based on PRISMA 2009, although the authors note that PRISMA was updated in 2020. Indeed, the PRISMA 2020 authors state "The PRISMA 2020 statement (including the checklists, explanation and elaboration, and flow diagram) replaces the PRISMA 2009 statement, which should no longer be used.". It is unclear to me why readers would be interested in reading the assessments of an outdated tool.

*Response: Your point is well-taken; we have provided clarity on the reasoning for use of PRISMA 2009 within the manuscript, including in the title of manuscript. The dataset used for this study included publications in the selected journals, published between 1993-2018. We therefore elected to use the PRISMA 2009 Statement as the reporting guideline by which to assess studies since it was the guideline published within the time span of the publication dates for the articles assessed. We had hoped to publish this article sooner and can only state that progress was delayed by personal and professional challenges during COVID, including an extended period of professional leave, between mid-2020 and early 2023. We hope the findings still have value within the broader context of understanding author uptake of reporting guidelines and how reporting guidelines affect reporting practices for published evidence syntheses. Anecdotally, in our role as medical librarians, we have observed resistance among authors involved in systematic reviews and meta-analyses in faithfully following PRISMA, even to the point where primary authors for reviews we have been involved in removing medical librarians’ search-related details from the review manuscript, with an insistence that readers will ‘neither understand nor care about granular details of search processes.’ In writing this, we realize that the cultural issues within science are beyond the scope of this review and are yet the likely strong drivers of level of uptake of and fidelity to the reporting guideline.*

*In thinking about how the use of PRISMA 2009 in this study might affect the usefulness of the results in light of the publication of PRISMA 2020, we checked the variables assessed for each study against both the QUORUM and PRISMA 2020 guidelines and note that many of the variables are applicable to all three reporting guidelines:*

*Title: Explicit identification of the report as a systematic review (or meta-analysis)*

*Eligibility: Specify the criteria used for study eligibility decisions (inclusion and exclusion)*

*Methods: Search: Provide details about the information sources and search methods [QUORUM], such that a search could be repeated [PRISMA 2009], for all databases [PRISMA 2020]*

*Methods: Study Selection: State the methods used for selecting studies (not included in QUORUM)*

*Methods: Risk of bias in individual studies: Describe methods used to assess risk of bias in included studies*

*Results: Study selection: Describe the results of the search and selection process, ideally using a flow diagram*

*For several of the sections of PRISMA, our variables correspond more closely to the specific requirements of PRISMA 2020 than those of PRISMA 2009, such as the reporting of the number of people involved in title and abstract screening and full text review and described use or non-use of blinding in study selection. In this way, we hope that the results of the study provide evidence of the importance of those additional, key details, as well as the levels of reporting for those items prior to the publication of PRISMA 2020.*

*In addressing your concerns within the manuscript, we added the following language to the manuscript:*

*“Because the dataset for this study included articles published in high-impact dental medicine journals between 1993-2018, the PRISMA 2009 checklist was used to assess reporting quality. . . The authors additionally note that several of these items are largely unchanged between the QUORUM, PRISMA 2009, and PRISMA 2020 checklists, such as the identification of the report as a systematic review in its titling, the specification of inclusion and exclusion criteria, and reporting study selection results for each phase of the review. Therefore, studies not meeting many of the assessed checklist items for PRISMA 2009 would typically fail to meet the same item for QUORUM and PRISMA 2020.” (p. 7)*

*We also added an acknowledgement of the limitations of the study within the context of the release of PRISMA 2020, with the need for further studies:*

*“As previously noted, many of the items assessed are included in the QUORUM, PRISMA 2009 and PRISMA 2020 checklists. A follow-up study to assess improvements in reporting for items uniquely and expressly required in PRISMA 2020 would help further elucidate awareness and meaningful use of PRISMA 2020 among authors of SRs/MAs.” (p. 35)*

*And in the Conclusion, we added:*

*“While conduct and reporting of research are distinct concepts, they are closely intertwined for systematic reviews [2]. This study of the implementation of PRISMA 2009 reporting guidelines in dental SRs and MAs indicates that there is a need for increased use of and education related to PRISMA, particularly following the publication of PRISMA 2020. We suggest that those who are conducting SRs in dental medicine should become familiar with the PRISMA 2020 Statement in order to understand the complex nature of the individual reporting items and the methodological concerns informing the reporting items, prior to the start of the review process. Author examination of the Explanation and Elaboration publication accompanying PRISMA 2020 [65] is highly recommended, beyond just using the checklist as a standalone item.”*

*We do hope that someone will undertake a follow-up study to assess improvements in reporting following the publication of the PRISMA 2020 statement. Between the effects of the pandemic on publication timelines for non-COVID research, along with generally slow diffusion of knowledge and adoption of guidelines, broadly writ, we hazard a guess that the number of systematic reviews in the selected journals that cite the PRISMA 2020 Statement remains small, though growing, at present. A rapid and (forgive us) non-systematic assessment of the citation data for the five records for the publication of the PRISMA 2020 Statement confirms this (as of November 14^th^, 2023, in Scopus): the Journal of Dentistry has 23 publications that cite the PRISMA 2020 Statement and the International Endodontic Journal has 22 publications citing PRISMA 2020, the rest of the journals we assessed have fewer (or none). With time, the number of reviews citing and applying PRISMA 2020 in these journals should increase, allowing a follow-up study.*

Reviewer #2: I must congratulate the authors for considering this research question. It is an important topic for evidence based dentistry researchers. There are a couple of suggestions that can improve the paper-
1. It is important to emphasize that quality of a systematic review is dependent on the methodological aspects and the analysis of data. This is assessed by tools such as AMSTAR-2 and ROBIS. Authors can discuss this and state that their research question was related to overall quality of reporting quality however it is important to recognize how the evidence based medicine has evolved in terms of quality.

*Thank you for your suggestions. We have added language to the introduction to address the importance of methodological quality assessment tools*

*“The methodological quality of systematic reviews and meta-analyses has been a long-standing concern, [9–12] with considerable effort from the Cochrane Collaboration and the Institute of Medicine, among others, invested in creating and updating methodological guidelines and recommendations to improve rigor. Likewise, tools to assess methodological quality of published evidence syntheses, such as AMSTAR (A MeaSurement Tool to Assess systematic Reviews) [13] and risk of bias assessments for systematic reviews, such as ROBIS [14] were developed to assess published evidence syntheses. The early concerns about the methodological quality of SRs/MAs were quickly succeeded by concerns related to the reporting quality thereof, with the understanding that, without thorough and transparent reporting, neither methodological rigor nor reliability of results and recommendations could be assessed.” (p. 3)*

*We additionally made our research question more clear in its focus on reporting quality:*

*“The authors sought to address four research questions related to the effect of PRISMA on* ***reporting*** [emphasis added here] *quality for SRs/MAs in Dental Medicine journals: …” (p. 5)*

*Lastly, we added to the Discussion section:*

*“While there is a relationship between the conduct of a study and its record [37], the PRISMA Statement is clear in its intended application as guiding the reporting of methodological details rather than the methodological conduct of the review. There are key differences in these two considerations. For example, while the guidelines’ requirement to report risk of bias assessment details may cue authors to undertake risk of bias assessment for included studies, that cue does not guarantee that the risk of bias assessment instrument was appropriately chosen or properly applied. Likewise, a reproducible search may neither be rigorous in its conceptualization, nor appropriately mapped across relevant databases. Such methodological quality assessments are better determined through assessment instruments such as AMSTAR [13] or ROBIS [14], and are the considerations that should ideally, ultimately drive the level of trust in the results and recommendations of the SR/MA.” (p. 33-34)*

2. They can touch bases on how the overview of systematic reviews and evidence mapping papers are changing the way evidence analysis has been done and the role PRISMA has played in making it possible.

In the discussion, we added:

*“This is increasingly important as umbrella reviews (including meta-umbrella reviews) are undertaken to better understand the benefits and harms of an array of interventions or exposures. Low quality reporting in the systematic reviews and meta-analyses meeting inclusion criteria in the umbrella then create additional difficulties for the team involved in the umbrella review.” (p. 32)*

Our thanks again for the reviewers’ comments. We hope that we have adequately addressed the comments and that our revisions reflect the improvements requested.

Respectfully,

Kerry Sewell and co-authors
